# Supplementary material for: Development of a computer-aided design software for the quantitative evaluation of aesthetic damage
Source: PLoS One. 2019 Dec 18;14(12):e0226322. doi: 10.1371/journal.pone.0226322 (PMC6919621; doi:10.1371/journal.pone.0226322)
Supplement: S1 Code site — 1. (ZIP) [file pone.0226322.s001.zip › [EESC_jr][Projeto]Relatorio_07_21/reports/templates_zip/gentelella-master/vendors/Flot/examples/basic-options/index.html]

Flot Examples: Basic Options


## Basic Options

There are plenty of options you can set to control the precise looks of your plot. You can control the ticks on the axes, the legend, the graph type, etc.

Flot goes to great lengths to provide sensible defaults so that you don't have to customize much for a good-looking result.

Copyright © 2007 - 2014 IOLA and Ole Laursen
